# Stress analysis in a layered aortic arch model under pulsatile blood flow

**DOI:** 10.1186/1475-925X-5-25

**Published:** 2006-04-24

**Authors:** Feng Gao, Masahiro Watanabe, Teruo Matsuzawa

**Affiliations:** 1 Graduate School of Information Science, Japan Advanced Institute of Science and Technology, 1-1 Asahidai, Nomi, Ishikawa, 923-1292, Japan; 2 Center for Information Science, Japan Advanced Institute of Science and Technology, 1-1 Asahidai, Nomi, Ishikawa, 923-1292, Japan

## Abstract

**Background:**

Many cardiovascular diseases, such as aortic dissection, frequently occur on the aortic arch and fluid-structure interactions play an important role in the cardiovascular system. Mechanical stress is crucial in the functioning of the cardiovascular system; therefore, stress analysis is a useful tool for understanding vascular pathophysiology. The present study is concerned with the stress distribution in a layered aortic arch model with interaction between pulsatile flow and the wall of the blood vessel.

**Methods:**

A three-dimensional (3D) layered aortic arch model was constructed based on the aortic wall structure and arch shape. The complex mechanical interaction between pulsatile blood flow and wall dynamics in the aortic arch model was simulated by means of computational loose coupling fluid-structure interaction analyses.

**Results:**

The results showed the variations of mechanical stress along the outer wall of the arch during the cardiac cycle. Variations of circumferential stress are very similar to variations of pressure. Composite stress in the aortic wall plane is high at the ascending portion of the arch and along the top of the arch, and is higher in the media than in the intima and adventitia across the wall thickness.

**Conclusion:**

Our analysis indicates that circumferential stress in the aortic wall is directly associated with blood pressure, supporting the clinical importance of blood pressure control. High stress in the aortic wall could be a risk factor in aortic dissections. Our numerical layered aortic model may prove useful for biomechanical analyses and for studying the pathogeneses of aortic dissection.

## Background

The aorta is the main blood artery that delivers blood from the left ventricle of the heart to the rest of the body, and many cardiovascular diseases, such as aortic dissection, often occur on the aortic arch. It has been well established that many diseases are closely associated with the flow conditions in the blood vessels [1] and the blood flow in the aortic arch has been widely studied in the past [2-4].

Fluid-structure interactions play an important role in the cardiovascular system. There have been many recent studies of fluid-structure interaction in the aortic valve [5], aortic aneurysm [6], and in stented aneurysm models [7]. However the interaction between a pulsatile blood flow and the aortic wall in an aortic arch model has not yet been studied.

From the mechanical point of view, ruptures appear if the stresses acting on the wall rise above the ultimate value for the aorta wall tissue [6]. Mechanical stress plays a crucial role in the functioning of the cardiovascular system; therefore, stress analysis is a useful tool for understanding vascular pathophysiology [8]. Thubrikar et al. [9] used finite element analysis to determine the stresses in an aneurysm of the aorta; they found that longitudinal stress in the bulb is the only stress that increases significantly and could be responsible for the tear in an aortic dissection. Stress analysis of the aortic arch was implemented to study the effects of aortic motion on aortic dissection [10].

The present study is concerned with the stress distribution in a layered aortic arch model with interaction between a pulsatile flow and the wall of the blood vessel. Circumferential and longitudinal stress, as well as composite stress in the wall plane, is presented.

## Methods

### Geometry and wall properties

**Figure 1:**
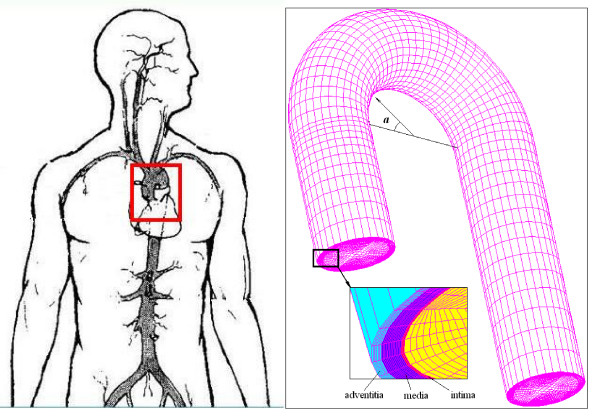
**The finite element model of the aortic arch**. The branches of the arch were neglected as a first order approximation. Angle ***a ***represents the wall position in the median longitudinal cross-section. The inset shows the thickness of each layer.

The radius of the arch was set at 30 mm [11] and the diameter of the vessel was assumed to be uniform (24 mm). The average diameter of the aorta is 20–25 mm [12]. The thickness of the whole wall was chosen to be 2 mm according to the characteristics of various types of blood vessels in [13]. The arch angle ***a ***is measured at the circumference of the median longitudinal cross-section, and ranges from 0° and 180° (Fig. 1), so that the wall position of the arch, as denoted by the arch angle, can be discussed.

An average thickness ratio of intima/media/adventitia of 13/56/31 for arteries was observed in Schulze-Bauer's studies [14]. The thickness ratio of media/adventitia is 2/1 in the computational model for the arterial wall presented by Driessen et al. [15]. In this three-layered wall model, the intima/media/adventitia thickness ratio was set to 1/6/3. Therefore, the thicknesses of the intima, media, and adventitia were ***t***_***i ***_= 0.2 mm, ***t***_***m ***_= 1.2 mm, and ***t***_***a ***_= 0.6 mm, respectively.

**Figure 2:**
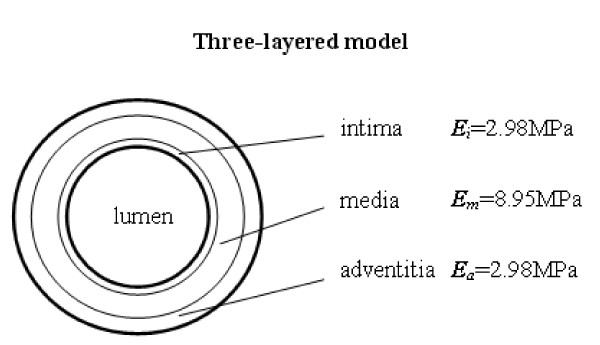
**Three-layered models**. The Young's modulus of layers is shown.

In Mosora's experiments [16], the Young's modulus of the thoracic ascending aorta was 2 MPa to 6.5 MPA. In vivo, the surrounding connective tissue and muscle can support the aortic arch. In this study, the aortic arch is a self-supporting structure. The maximum ***E ***= 6.5MPa was chosen for the Young's modulus of the aorta wall. Xie et al. [17] performed bending experiments and showed the Young's modulus of the inner layer (intima and media) was three to four times larger than that of the outer layer (adventitia), and we can deduce from Fischer's experimental data [18] that the Young's modulus of the intima is smaller than that of the media. In the present study, the Young's modulus of the media is assumed to be three times larger than that of the adventitia and the intima. Since the mean Young's modulus of the vessel wall across whole wall volume is invariable, we assume that the Young's modulus of each layer is in inverse proportion to the area of the layer in the cross section. Based on the area equation, the Young's modulus of the intima, media, and adventitia layers is 2.98MPa, 8.95MPa, and 2.98MPa respectively. Fig. 2 shows the three-layered aortic wall.

### Fluid-structure interaction

Fig. 1 shows the circulatory system; the blowup is the FEM model of the aorta. The finite element analysis model of the aorta was made of 38400 eight-node brick elements for the solid domain and 26880 eight-node brick elements for the fluid domain. There are 20 elements through the thickness of the aorta wall.

In a fluid-structure interaction problem, fluid affects structure and structure also affects fluid. In this study, the procedure is based on the loose coupling of three fields of problems: the flow, the elastic body, and the mesh movement – that is, CFD (computational fluid dynamics), CSD (computational structural dynamics), and CMD (computational mesh dynamics) procedures [19].

**Figure 3:**
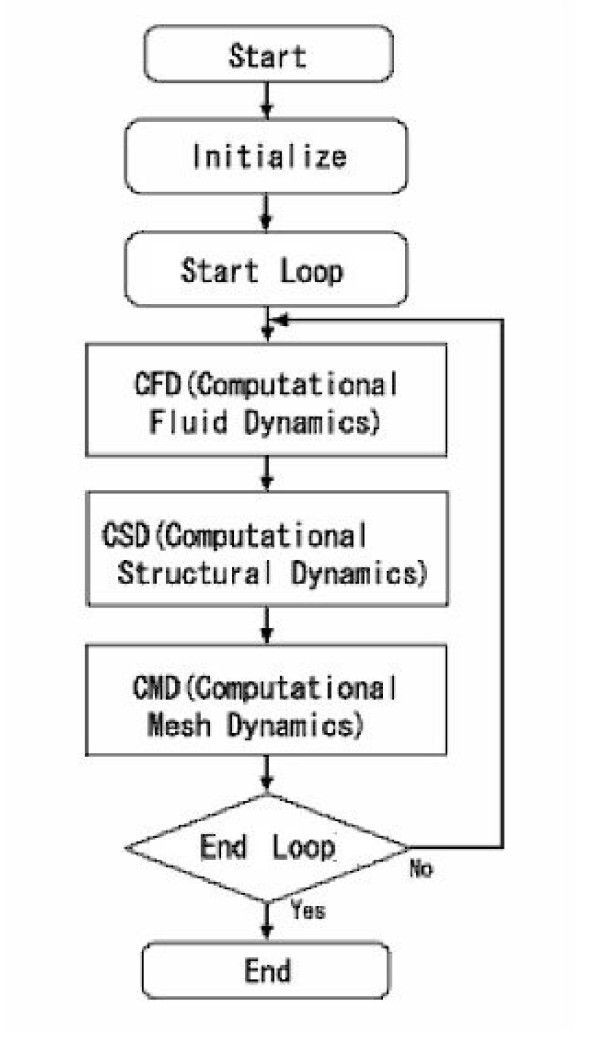
Strategy of the computational procedure.

The FSI (Fluid Structure Interaction) algorithm treats the equations that solve the fluid problem, the structural problem, and the re-meshing problem by a staggered approach; that is to say, the algorithm solves the equations in sequence. The overall computational procedure adopted to solve FSI problems is depicted in Fig. 3. Operatively, a ALE (Arbitrary Lagrangian Eulerian) algorithm is used which seeks, at each step increment, the convergence of the three blocks of equations, fluid (CFD), solid (CSD) and mesh movements (CMD). These must then converge altogether before a new step is initiated. The detailed equations are described in the Appendix.

The code *Fidap *(Fluent Inc., Lebanon, NH) was used to carry out the simulation. Bar-Yoseph et al. [20] gave many examples to demonstrate the validity of the method used in this study. Marzo et al. [21] also used the same method to do a FSI simulation and got excellent results.

Typically, 160–190 iterations were required per time step to reduce the residuals. The numerical convergence is a relative change in the solution from one iteration to the next of less than 0.0001.

Circumferential stress is the normal stress that points along the tangent direction to a cross-section circle in the cross-section plane. Longitudinal stress is the normal stress that points in the direction of the axis of the vessel. Composite stress is the composition of the circumferential stress vector and the longitudinal stress vector, which is the resultant stress in the wall plane.

### Boundary condition and properties of fluid

**Figure 4:**
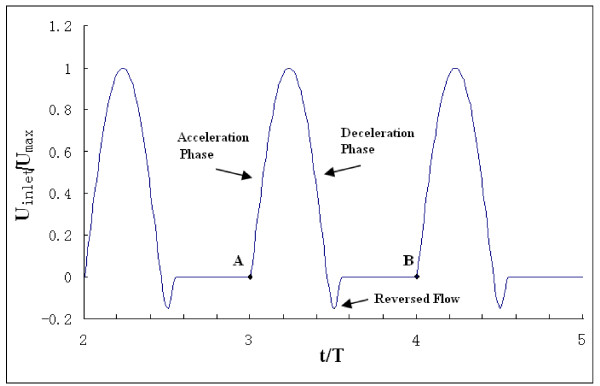
**Inlet velocity profile**. t is the time, T is the total time in one cycle. For the final run, the cardiac cycle starts at A and ends at B.

The boundary conditions are time-dependent. At the aortic inlet, a flat flow velocity profile was used together with a pulsatile waveform based on experimental data reported by Pedley [22]. This waveform is shown in Fig. 4. The assumption of a flat velocity profile at the aortic inlet is justified by in vivo measurements using hot film anemometry on various animal models, which have demonstrated that the velocity profile distal to the aortic valve are relatively flat [23]. The Reynolds number for blood flow is approximately 4000 in the aorta [24]. In our calculations, the Reynolds number is fixed at Re = 4000 based on the inlet velocity at peak systole of the cardiac cycle. There is pressure both at the inside and the outside of the blood vessel and the pressure difference between the inside and the outside of the blood vessel tends to deform the blood vessel [24]. This study was concerned mainly with the effect of the relative pressure on the wall stress. At the aortic outlet, a zero pressure condition was applied. The surface of the inlet was fixed. The outer edge of the outlet was constrained in the axial direction and permitted to move in the other directions. There was no constraint for the other parts of the aorta model.

The simulation reached nearly steady-state oscillation after approximately the third cycle. The fourth cycle was used as the final periodic solution and it is presented in this paper.

The fluid is Newtonian with a density of 1050 kg/m^3 ^and a viscosity of 0.0035 Pa s. Blood is essentially a suspension of erythrocytes in plasma and shows anomalous viscous properties at low velocities. However, the Newtonian assumption is considered acceptable since minor differences in the basic flow characteristics are introduced through the non-Newtonian hypothesis [25].

## Results

### Result pressure

**Figure 5:**
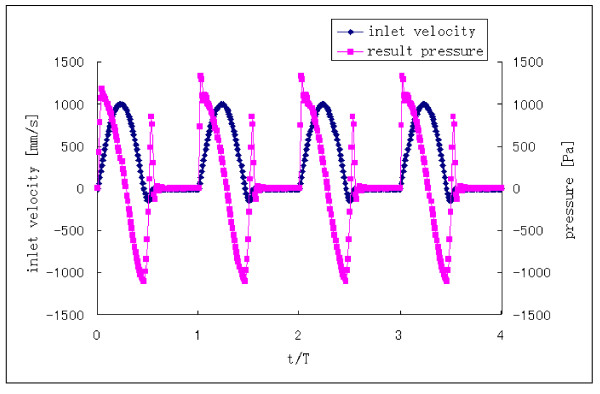
**Pressure waveform at inlet corresponding to inlet velocity**.

The pressure waveform at the inlet corresponding to the inlet velocity was extracted from result data (Fig. 5). We see the pressure rises above zero (1330Pa) at the start of systole during the accelerative phase and then becomes negative at peak systole, reaching a minimum value (-1100Pa) at the max reverse flow. There is a transient increase in pressure after the max reversed flow phase. A negative pressure represents the decelerative phase of the aortic flow.

**Figure 6:**
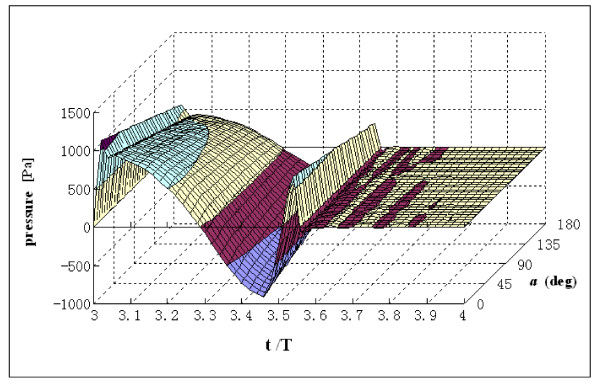
**Pressure at centerline as a function of the function of arch angle *a *and time t/T**. End diastole is at t/T = 4.0. Angle ***a ***represents the wall position in the arch portion.

Fig. 6 shows the variations of pressure along the arch at centerline during the cardiac cycle. The variations of pressure as a function of time are similar to the results shown in Fig. 5. Positive pressure values decrease along the arch portion.

### Variations of stresses along arch during cardiac cycle

**Figure 7:**
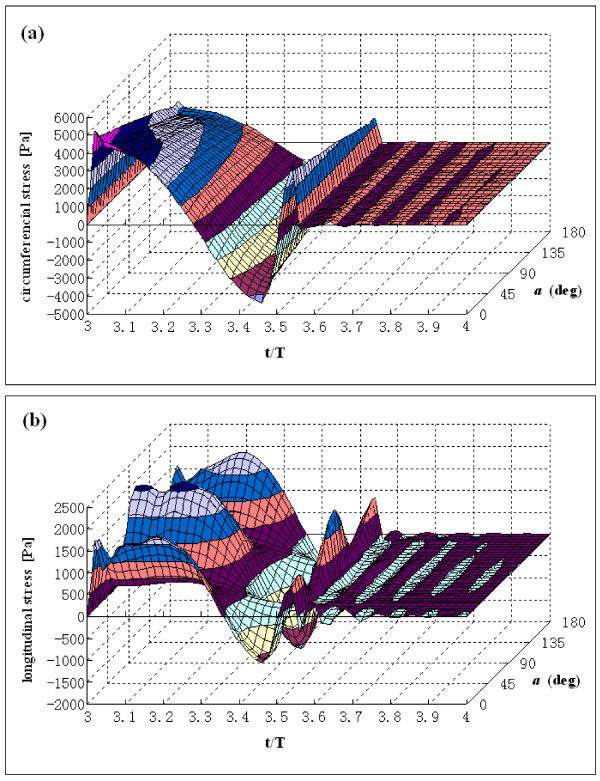
**Variation of circumferential and longitudinal stress at arch outer wall as a function of the function of arch angle a and time t/T**. (a) Circumferential stress variation. (b) Longitudinal stress variation. End diastole is at t/T = 4.0. Angle a represents the wall position in the arch portion.

Tears and dissections usually involve the outer wall of the arch [26], so the stress distribution in the outer wall was investigated. Fig. 7 presents the variations of circumferential stress and longitudinal stress on the outer wall along the arch portion during the cardiac cycle. The stresses are averaged across the aortic wall thickness.

The circumferential stress on the outer wall initially increases at the start of systole and then decreases below zero during the deceleration phase. After the reverse flow, there is a transient increase in mean circumferential stress. The circumferential stress is higher in the ascending portion than in the descending portion. The variation of the mean circumferential stress in the outer wall of the arch is similar to the variation of the pressure at the arch outer wall.

Longitudinal stress initially increases at the acceleration phase of systole, then deceases, and then increases a little at the reverse flow. The high stress regions along the arch are at the entrance to the ascending portion, the top of the arch, and the distal end of the arch.

**Figure 8:**
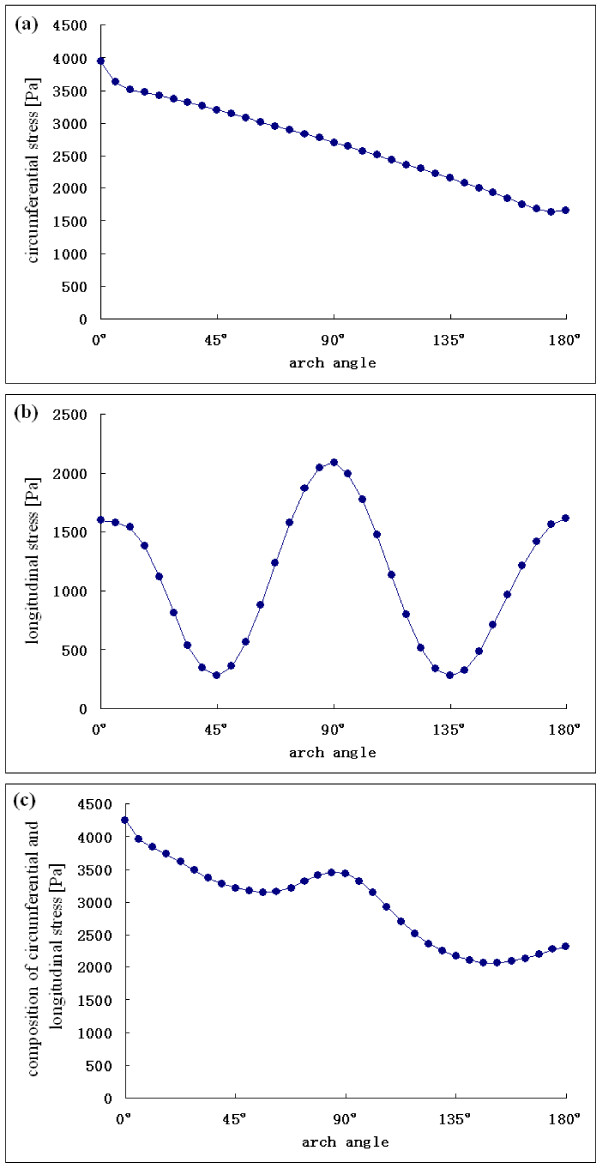
**Stress distribution on the outer wall along the arch portion**. (a) Circumferential stress distribution. (b) Longitudinal stress distribution. (c) Composite stress distribution.

Since the stress in the acceleration phase is high, a transient time t/T = 0.12 in the middle of the acceleration phase was selected to characterize the variations of stresses along the arch in detail. The circumferential stress and the longitudinal stress, as well as the composite stress along the arch at selected transient times, are shown in Fig. 8.

The circumferential stress decreases along the arch. The longitudinal stress along the arch gets peak values at the entrance to the ascending portion, the top of the arch, and the distal end of arch. The composite stress decreases, with a peak at the top of the arch along the arch portion.

### Variations of stresses across the wall

Four positions, ***a ***= 22.5°, ***a ***= 67.5°, ***a ***= 112.5°, and ***a ***= 157.5°, were selected in the arch portion to illustrate the variation of stresses across the wall.

**Figure 9:**
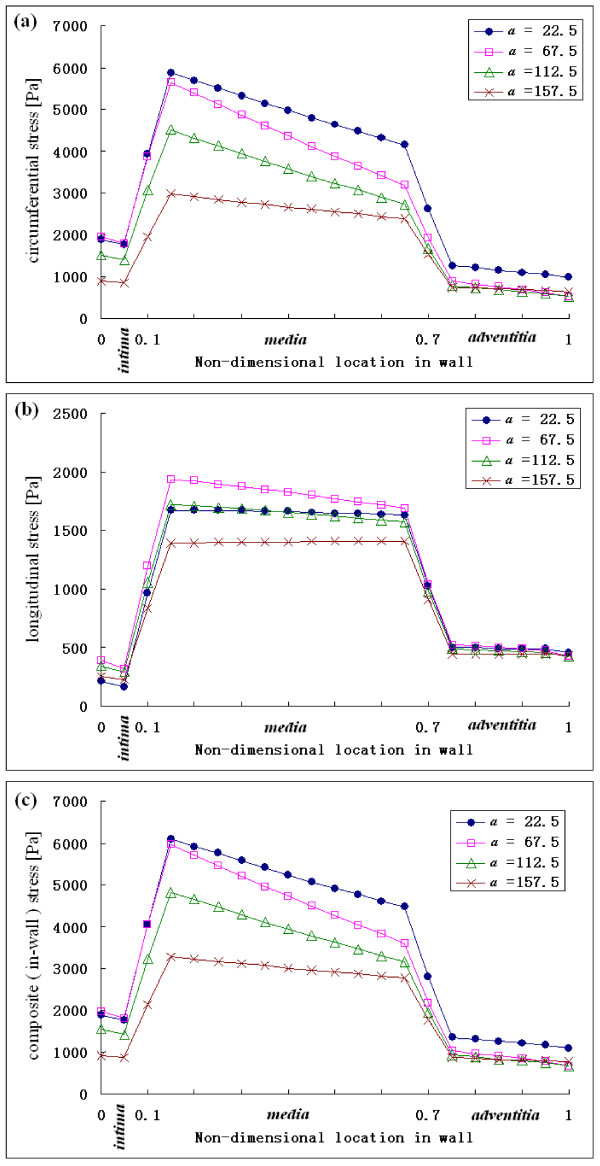
Variation of the stresses across the wall at four positions (*a *= 22.5°, *a *= 67.5°, *a *= 112.5°, and *a *= 157.5°) at selected time of systolic acceleration (t/T = 3.12).

The variations of circumferential, longitudinal, and composite stress across the three-layer model wall at four positions at systolic acceleration (t/T = 3.12) are shown in Fig. 9. The stress is much higher in the media layer than in the intima and adventitia layers.

## Discussion

We have simulated the complex mechanical interaction between blood flow and wall dynamics by means of computational coupled fluid-structure interaction analyses and shown the results for mechanical stress in the layered aortic arch model. The composite stress in the aortic wall plane is high at the ascending portion and along the top of the arch and is higher in the media than in the intima and adventitia across the wall thickness.

Circumferential stress was considered an important parameter for mechanosensitive receptors. Under a pressure *P*, the circumferential and longitudinal stresses in a cylinder of thickness *t *and radius *R *are *PR/t *and *PR/2t*, respectively [9]. Our resulting pressure waveform at the inlet in the present study agrees with the results in Tyszka's [27] paper. This demonstrates that circumferential stress depends on the systolic blood pressure. Furthermore, this explains the fact that 70–90% of patients with aortic dissection have high blood pressure [26,28-30] and supports the clinical importance of blood pressure control in reducing the risk of tearing and rupturing in aortic dissection.

The pressure P decreased along the arch portion, and the radius R and thickness t did not change very much. However, the longitudinal stress depicted in Fig. 8(b) along the arch gets peak values at the entrance to the ascending portion, the top of the arch, and the distal end of arch, so the variation would be unpredictable using *PR/2t; *thus, the shape of the arch and fluid force may play crucial roles in aorta mechanics. Similar work on the stress distribution on the aortic arch has been presented by Beller et al. [10], in which circumferential and longitudinal stress were found to be maximum at the top of the arch. Unfortunately, they only used a load condition of 120 mmHg luminal pressure and the pulsatile flow condition was not considered. In recognizing the influential effects of pulsatile flow in the aortic mechanism, a more realistic wall stress distribution was simulated in our coupled model. Bellers et al. [10] included branch vessels in their model. Our study was concerned mainly with the effects of the aortic arch on stress distribution. Therefore, the branches along the top of the aortic arch were not included in the model.

Thubrikar et al. [9] have proposed that longitudinal stress could be responsible for transverse tears in the aortic dissection. Roberts [26] reported that in 62% of patients the tear is located in the ascending aorta, usually about 2 cm cephalad to the sinotubular junction. This means the location is about ***a ***= 0°, at the entrance to the ascending portion of the arch. After the ascending aorta, about 20% of tears are at the aortic isthmus portion [26], which is near the top of the arch. In the present study, the longitudinal stress achieved peak values at the entrance to the ascending portion and the top of the arch. Yet, the longitudinal stress also peaked in the distal end of the arch, and tears do not often occur at this location.

Composite stress was high at the entrance to the ascending portion and at the top of the arch; the value at the entrance to the ascending portion was a little higher. The location of this high composite stress value is consistent with the tear locations identified in the Roberts report [26]. This implies that both circumferential and longitudinal stress may contribute to the tear in an aortic dissection and high composite stress in the aortic wall could be a risk factor for tears in aortic dissections.

The stress variation across wall thickness was due to the non-homogeneous wall properties. Our results showed that the stress was highest in the media across the aortic wall thickness and this agreed with the results of Maltzahn et al. [31], that the media was subject to much higher stresses. These results might partially explain why the location of the dissection is in the aortic media [26].

Previous studies [32,33] examined the properties of the aorta and showed the aortic wall is anisotropic. The smooth muscle cells in aortic wall were torn loose from their attachments to each other and to the adjacent elastin [33], so the dissection may propagate rather than tearing back through the intima or out through the adventitia. Residual stresses can affect stress distribution through the arterial wall. Circumferential stress distributions including residual stress have shown decreased inner wall stress [34], which may be induced by the negative value of residual stress in inner side of wall [17]. The residual stress is high in the outer side of media and positive at adventitia [17] and this may increase the stress at the outer side of media and at adventitia.

The aortic arch model used here only partially simulated the real situation because it neglected the effect of the branches of the arch [3,4] and the aortic arch's non-planarity [35]. Because this was an initial attempt; the model was purposely kept simple in order to give an insight into the stress distribution on the aortic arch with interaction between a pulsatile flow and the wall. Furthermore, more than 80% of tears and dissections are at the aorta, not at branches of the arch [26,36]. In the future investigations, the branches will be added to the model. The mechanical properties of arteries have been reported extensively in the literature. The type of nonlinearity is a consequence of the curvature of the strain-stress function, which shows that an artery becomes stiffer as the distending pressure increases. Horsten et al. [37] showed that elasticity dominates the nonlinear mechanical properties of arterial tissues. In numerical simulation of the biomechanics in arteries [6-8,10], the wall was assumed to be an elastic constitutive model. Of course, a non-linear constitutive model could provide more biological aspects of the biomechanics. In future work we plan to implement the nonlinear properties of aortic wall within the code by means of user-subroutines.

## Conclusion

In summary, our analysis indicates that circumferential stress in the aortic wall is directly associated with blood pressure, supporting the clinical importance of blood pressure control. High stress in the aortic wall could be a risk factor for tearing in aortic dissections. This numerical layered aortic model may prove useful for biomechanical analyses and for studying the pathogeneses of aortic dissection.

## Appendix

Detailed equations of FSI algorithm

1. Computational Fluid Dynamics (CFD)

The ALE (Arbitrary Lagrangian Eulerian) form of the Navier-Stokes equations are used to solve for fluid flow for FSI problems. After spatial discretization by the finite element method, the Navier-Stokes equations are expressed in the ALE formulation:

**Ma **+ **N**(**v **- )**v **+ **Dv **- **Cp **= **f **    in ^*F *^Ω(*t*)     (1)

**C**^**T **^**v **    in ^*F *^Ω(*t*)     (2)

where **M **represents the fluid mass matrix; **N**, **D**, and **C **are, respectively, the convective, diffusive, and divergence matrices; **f **is an external body force; vectors **a**, **v**, and **p **contain the unknown values of acceleration, velocity, and pressure, respectively;  is the mesh velocity, calculated using Eq. (13) in CMD; and ^*F *^Ω(*t*) is the moving spatial domain upon which the fluid is described.

The following compatibility conditions are imposed on the interface between fluid and structure:



where (^*F*^) indicates values related to nodes placed in the fluid; (^*S*^) indicates values related to the nodes placed in the structure; and ^*I*^Γ(*t*) is the interface between fluid and structure at time *t*. ^*s *^**v **and ^*s*^**a **are calculated at the previous iteration loop in CSD.

The fluid velocity and pressure field are solved after initial conditions, boundary conditions and compatibility conditions are imposed on the fluid domain. For calculating the traction at the interface between fluid and structure vectors **a**, **v**, and **f **are decomposed into:



where (^*U*^) indicates values related to nodes on the velocity boundary, (^*I*^) indicates values related to the fluid nodes on the interface between fluid and structure, and the symbol (^-^) denotes the prescribed values. The load applied by the fluid on the structure along the interface between fluid and structure is obtained according to partitioned Eq. (1):



where ^*I *^**f **is the external force vector at the interface applied by the structure on the fluid; (^*IF*^) indicates values related to interface nodes related to the fluid; (^*IU*^) indicates values related to the interface nodes related to the velocity boundary; (^*II*^) indicates values related to the interface nodes only related to the interface;  is the traction applied by the fluid on the structure along the interface; **M**, **N**, **D**, **C, a**, **v**, and **p **are known values solved in the previous process in this equation. The traction  is imposed on the structure in CSD.

2. Computational Structural Dynamics (CSD)

The overall structural behavior is described by the matrix equation:

^*s *^**M **^*s *^**a**+^*s *^**K **^*s *^**d**= +  - ^σ ^**f **    in ^*s*^Ω(*t*)     (6)

where ^*S *^**M **and ^*S *^**K **are the structural mass and nonlinear stiffness matrices;  is the external body force vector; , calculated using the Eq. (5) in CFD, is the traction applied by the fluid on the structure along the interface; ^σ ^**f **is the force due to the internal stresses at the most recently calculated configuration; ^*S *^**d **is the vector of increments in the nodal point displacement; ^*S *^**a **is the vector of nodal point acceleration; ^*s *^Ω(*t*) is the structure domain at time *t*.  and ^σ ^**f **are equal to zero in this study. The equation of motion can be integrated and the displacement, velocity, and acceleration vectors can be calculated.

The displacement at the interface between fluid and structure can be calculated and the surface location is updated:

^*I *^**d **= ^*s *^**d **    on ^*I *^Γ(*t*)     (7)

The mesh displacement can be obtained at the interface between fluid and structure:

 = ^*I *^**d **    on ^*I *^Γ(*t*)     (8)

The mesh displacement  is imposed at the interface between fluid and structure in CMD.

3. Computational mesh dynamics (CMD)

An elasticity-based meshing algorithm was employed to solve the remeshing problem. The mesh was treated as a pseudo-elastostatic medium and the same algorithm Eq. (6), which was used to calculate the displacement of the structural body, was employed. In the algorithm, the mesh is modelled as a pseudo-elastic structure the deformation of which is based on the boundary condition resulting form Eq. (8) of the structural problem. Such displacements are obtained from



where  is the stiffness matrix, and  is the mesh displacement vector defined by



where  s the mesh position vector, and *t *_*ref *_is the reference time. Eq. (9) can be rewritten in a partitioned form as



On the interface between fluid and structure, the mesh displacement given by Eq. (8) from CSD is imposed. So we obtain



which shows that the fluid mesh motion is driven by the elastic body motion. This equation, together with the other boundary condition, was solved for the fluid mesh displacement.

The fluid mesh displacement was solved and the mesh geometry was updated. The mesh velocity field can be obtained:



The mesh velocity is imposed in CFD.
